# Supplementary figures and images for: Decolorization of azo dyes by a novel aerobic bacterial strain Bacillus cereus strain ROC
Source: PLoS One. 2022 Jun 15;17(6):e0269559. doi: 10.1371/journal.pone.0269559 (PMC9200318; doi:10.1371/journal.pone.0269559)

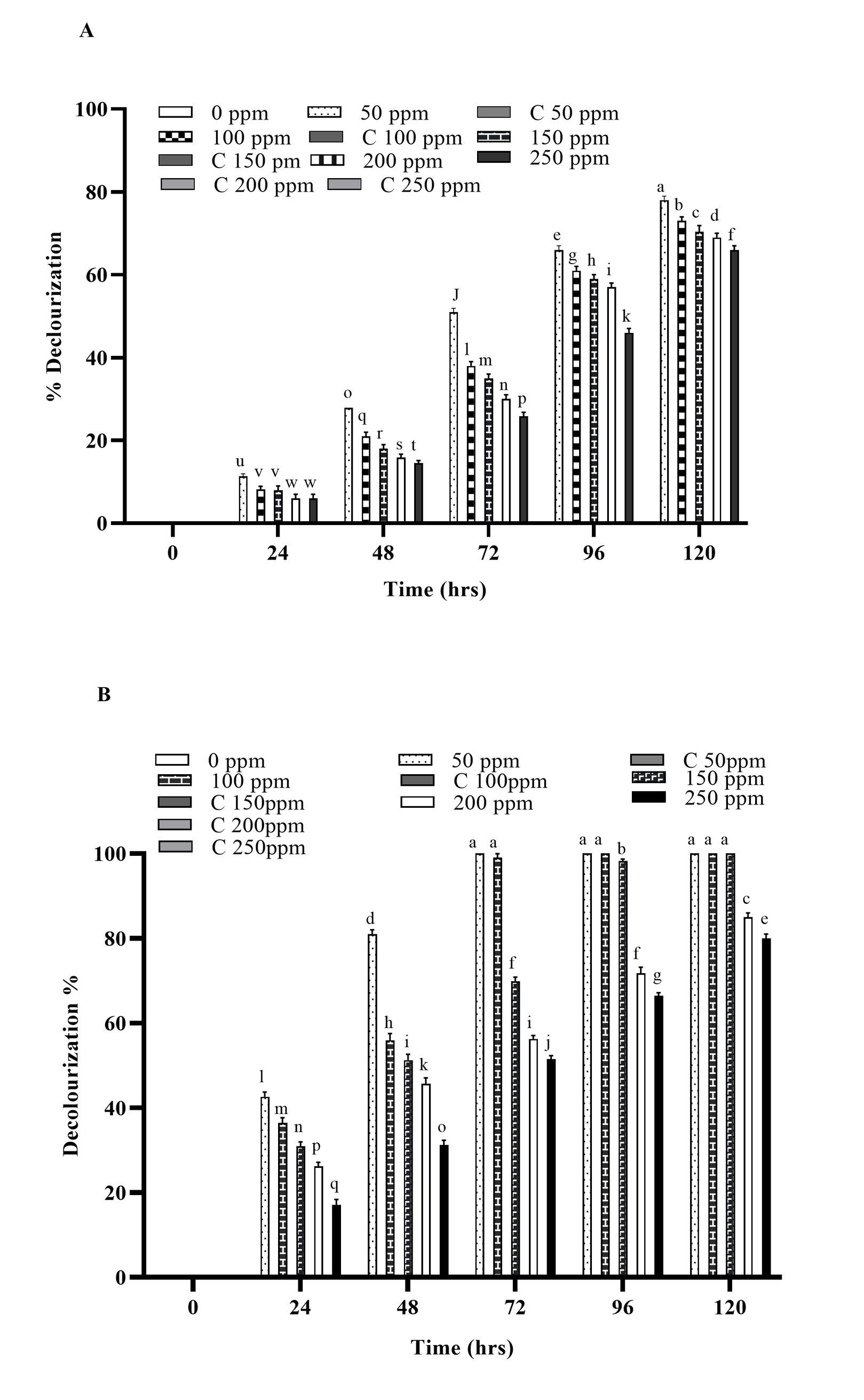

Supplement: S1 Fig — Decolourization of Reactive orange 16 by free and immobilized cells of bacterial strain (A) by free cells at 30 ˚C (B) by immobilized cells at 30 ˚C. (TIF) [file pone.0269559.s001.tif]

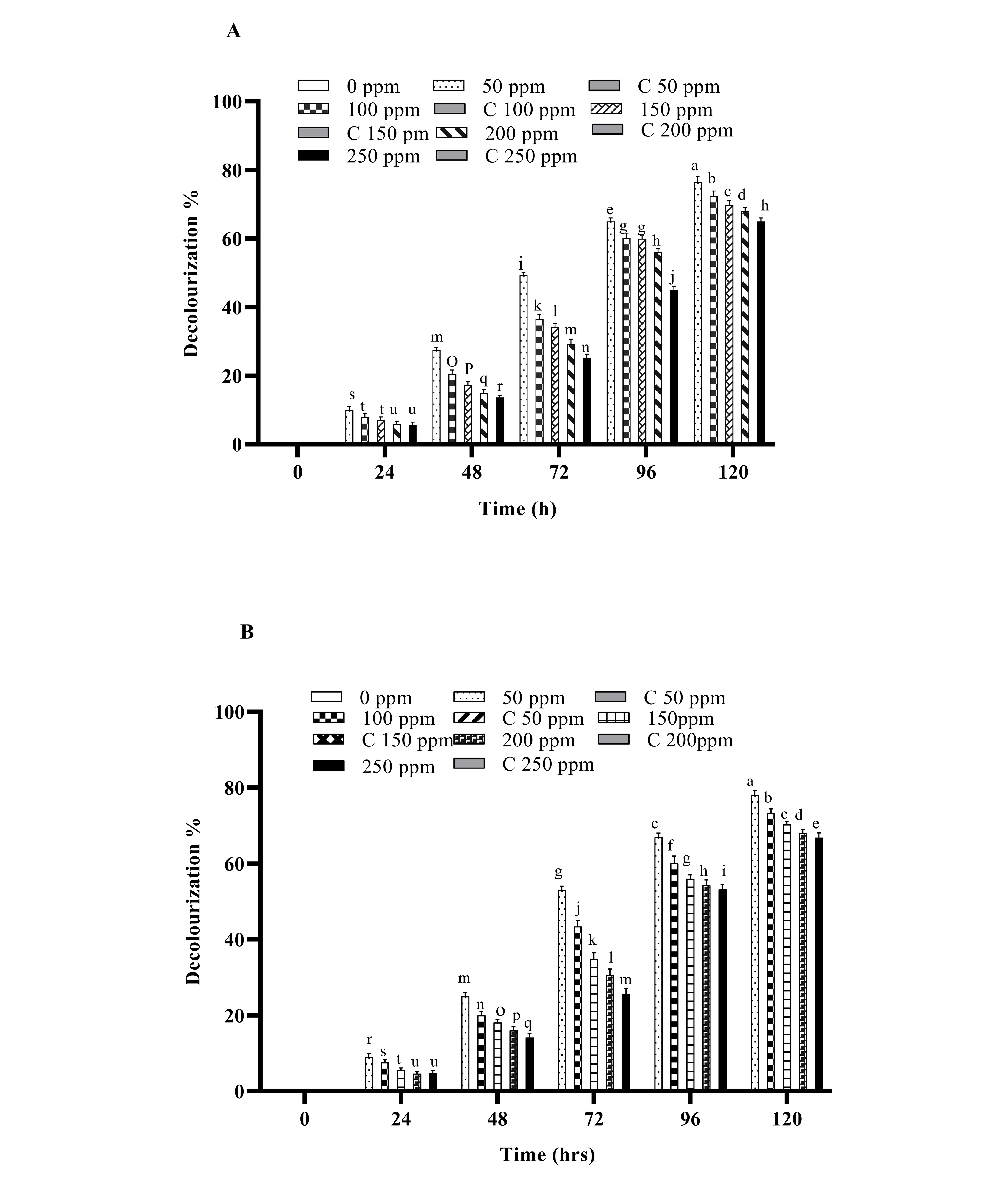

Supplement: S2 Fig — Decolorization of Reactive black 5 by free and immobilized cells of bacterial strain (A) by free cells at 30 ˚C (B) by immobilized cells at 30 ˚C. (TIF) [file pone.0269559.s002.tif]

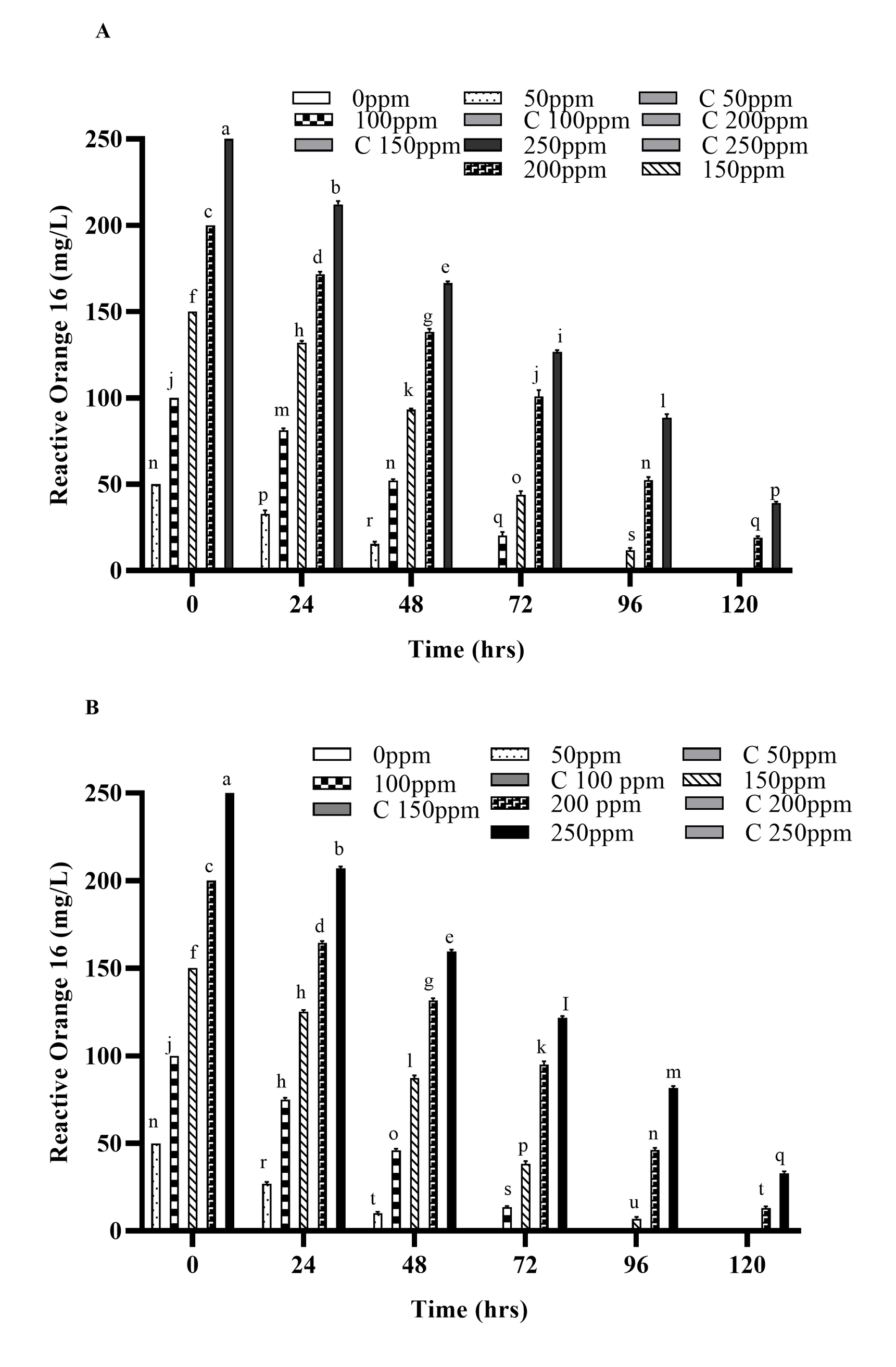

Supplement: S3 Fig — Concentrations of Reactive orange 16 degraded after treatment with free and immobilized cells of the bacterial strain (A) Degraded by free cells at 30 ˚C (B) Degraded by immobilized cells at 30 ˚C. (TIF) [file pone.0269559.s003.tif]

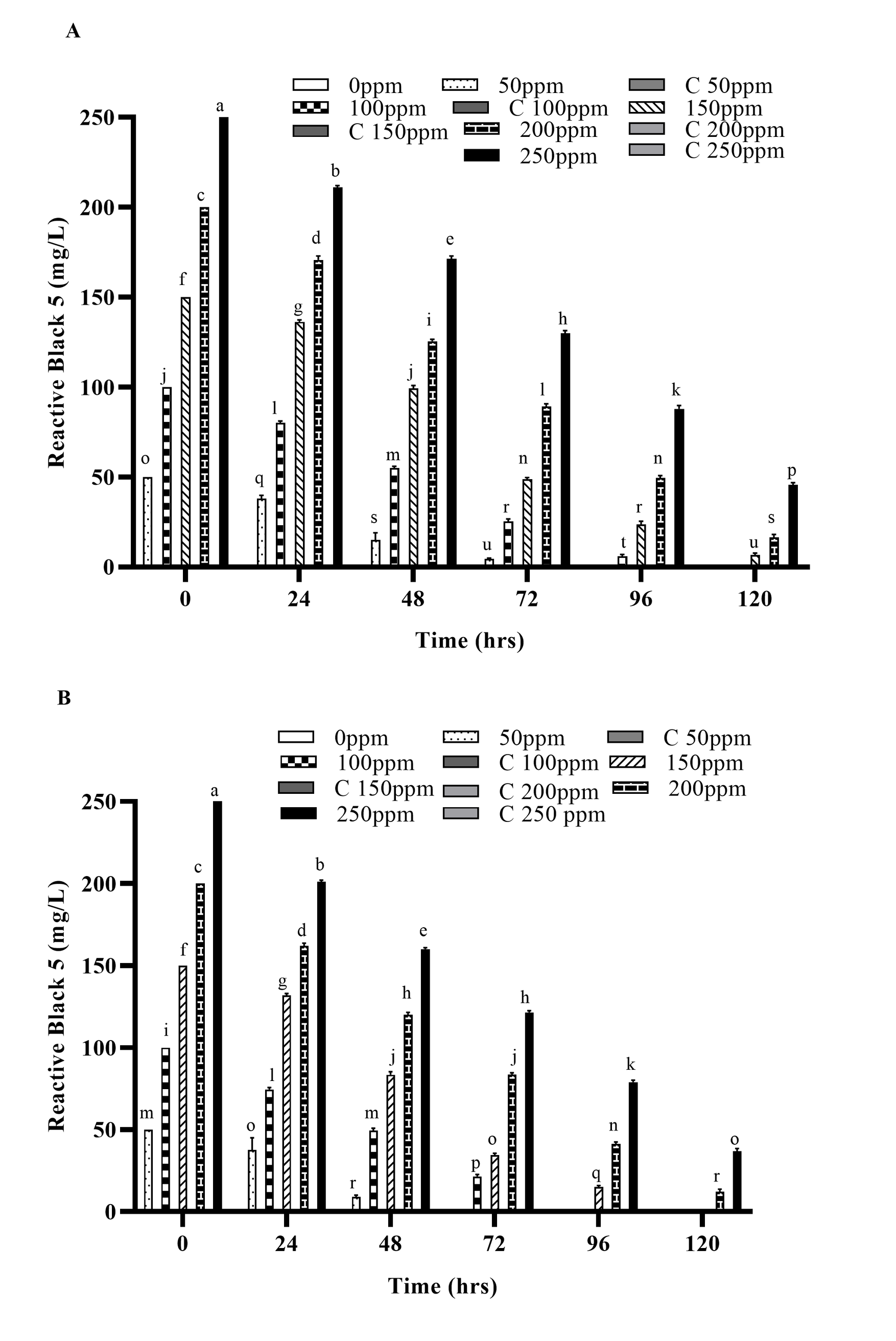

Supplement: S4 Fig — Concentrations of Reactive black 5 degraded after treatment with free and immobilized cells of the bacterial strain (A) Degraded by free cells at 30 ˚C (B) Degraded by immobilized cells at 30 ˚C. (TIF) [file pone.0269559.s004.tif]
