# Supplementary material for: Decolorization of azo dyes by a novel aerobic bacterial strain Bacillus cereus strain ROC
Source: PLoS One. 2022 Jun 15;17(6):e0269559. doi: 10.1371/journal.pone.0269559 (PMC9200318; doi:10.1371/journal.pone.0269559)
Supplement: Attachment — Submitted filename: Response to reviewers (Reviewer 1).docx [file pone.0269559.s006.docx]

Respond to Reviewers

1. **Please ensure that your manuscript meets PLOS ONE's style requirements, including those for file naming. The PLOS ONE style templates can be found at** [**https://journals.plos.org/plosone/s/file?id=wjVg/PLOSOne_formatting_sample_main_body.pdf and https:/**](https://journals.plos.org/plosone/s/file?id=wjVg/PLOSOne_formatting_sample_main_body.pdf%20and%20https:/)[**/journals**](/journals)**.plos.org/plosone/s/file?id=ba62/PLOSOne_formatting_sample_title_authors_affiliations.pdf**

Manuscript was checked thoroughly and follow the instructions provided by the journal site.

2. **We suggest you thoroughly copyedit your manuscript for language usage, spelling, and grammar. If you do not know anyone who can help you do this, you may wish to consider employing a professional scientific editing service**

Professor Colin Jackson who is a coauthor in this research article, has reviewed this manuscript for scientific as well as for English errors, whose native language is English.

3. **In your Methods section, please provide additional location information about your sampling sites, including geographic coordinates for the data set if available.**

The required information is given on Page #7, line # 145-146.

4. **In your Methods section, please provide additional information regarding the permits you obtained for the work. Please ensure you have included the full name of the authority that approved the field site access and, if no permits were required, a brief statement explaining why.**

The information is given on page #7, line # 146-148.

**Reviewer #1: The manuscript titled "Decolorization of azo dyes by a novel bacterial' has been reviewed and I found it an interesting piece of research; however, a lot of flaws have been indicated and are necessary to be taken care of before the further consideration.**

**Comments:**

**1.** The title of the paper is not complete ‘bacterial isolate………..name…..?? What is the novel in the bacterial strain, a huge number of bacillus species have been reported to decolorize the dyes. The word Novel is confusing here.

**Answer:** The name of the bacterial isolate is added in the title Page # 1, 2, line # 2, 23. The word novel is used for this strain as it decolorize the dyes aerobically.

**2.** Abstract………azo dyes…not azo- dyes

**Answer:** It has been corrected in the abstract. Page # 2, line # 29

**3.** Introduction contain a lot of grammar and structural mistakes. Please take care of…

**Answer:**

Methodology

**4.** All chemicals and reagents of analytical grade…..this is not a complete sentence

**Answer:** The sentence has been revised. Page # 7 , line #119

**5.** What is the rational to select 37 C temperature and why the aerobic conditions were provided especially when it is established that the azo dye decolorization is an anaerobic process?

**Answer:** Optimization of azo dyes degradation was done at various temperature i.e., 25,32,37ºC and 45ºC. but maximum decolorization with short period of time was observed at 37ºC.

Although the azo dyes are extremely stable under aerobic conditions and the spontaneous reduction of the azo bond under anaerobic conditions leads to highly carcinogenic and mutagenic colorless aromatic amines so the present study focused on the degradation of azodyes under aerobic conditions.

**6.** 16S rRNA gene…should be written correctly….Which primers were used?.....the mention the protocol description

**Answer:** The protocol has been described. Page # 8, Line #143, 144, 145

**7.** Phylogenetic tree should be correctly drawn. All the bacterial strain should be italicized. The isolated bacterial strain should be indicated as bold or red. A control group should be added in the tree

**Answer:**

**8.** In decolorization graphs, if decolorization was not measured at 0, then 0 time should not be added in the graphs. There is no need to add 0

**Answer:** The decolorization was also measured at 0, that’s why the 0 time is added in the graphs.

**9.**There should be an accession number of the identified strain

**Answer:** The accession number of the strain has been added. Page # 12, Line # 232.

**10.** The discussion is all over the place. The mere repetition of results should not be the part. Discussion should logically narrate the results by comparting with relevant literature.

**Answer:** Discussion has been improved as according to the suggestions made by the reviewer (Section: Results and Discussion). But as the section is Results along with discussion so we make try our best to not repeat results during discussion and solely discuss results after showing the results of the experiments.

**11.** The manuscript should be checked with the native English speaker to improve the grammar, syntax, sentence structure etc.

**Answer:** Professor Dr. Colin Jackson who belongs to Australia (Native English speaker) has checked the whole manuscript and improve the language as well as technical errors if there is any. He has read the manuscript thoroughly and incorporate the changes as shown **in Revised Manuscript with Track Changes**
